# Role of α7nAChR-NMDAR in sevoflurane-induced memory deficits in the developing rat hippocampus

**DOI:** 10.1371/journal.pone.0192498

**Published:** 2018-02-05

**Authors:** XiaoHong Tang, YiZe Li, JiYing Ao, Ling Ding, Yang Liu, Yuan Yuan, ZhiFen Wang, GuoLin Wang

**Affiliations:** 1 Department of Anesthesiology, Tianjin Medical University General Hospital, Tianjin, P. R. China; 2 Tianjin Research Institute of Anesthesiology, Tianjin, P. R. China; 3 School of Biomedical Engineering, Tianjin Medical University, Tianjin, P. R. China; Bilkent University, TURKEY

## Abstract

Detrimental effects of volatile anaesthetics, including sevoflurane, on the structure and function of the developing brain have been reported. The internalization of N-methyl-D-aspartate receptors (NMDARs) contributes to anaesthetic neurotoxicity. Both nicotinic acetylcholine receptors (nAChRs) and NMDAR play a critical role in the development of the nervous system. Moreover, nAChR can interact with NMDAR, and previous studies have demonstrated modulation of NMDAR by nAChR. In our study, we used an α7 nicotinic acetylcholine receptor (α7nAChR) agonist and α7nAChR antagonist to explore the role of α7nAChR and NMDAR in sevoflurane-induced long-term effects on memory and dendritic spine both in vivo and in vitro. The results revealed that the activation of α7nAChR attenuated the development of sevoflurane-induced memory deficit and dendritic spine changes, which might be by regulating NR2B-containing NMDAR trafficking from the intracellular pool to the cell surface pool in the hippocampus. Moreover, we demonstrated that α7nAChR could regulate NR2B-containing NMDAR via Src-family tyrosine kinase (SFK). Thus, our current study indicates that the trafficking of NR2B-containing NMDAR is regulated by α7nAChR via SFK in neonatal rat hippocampus, which may be secondary to sevoflurane-induced cognitive deficits in the developing hippocampus.

## Introduction

The developing brain is vulnerable to general anaesthetic exposure, especially during critical stages, such as the peak of synaptogenesis that occurs during—the first two weeks after birth in rats, and from the 6th month of gestation to a few years after birth in human [1]. Clinical studies have shown that anaesthesia and surgery exposure before age 3 may lead to learning disabilities and may increase the possibility of developmental delay or behavioural problems [2]. Similarly, sevoflurane, which is the most prevalently used inhalational anesthetic for paediatric patients, has been reported to lead to such impairments in animals [3–4]. Moreover, in humans, sevoflurane has been suggested to block the emotional memory [5]; however, the mechanism remains unclear.

Both nicotinic acetylcholine receptors (nAChRs) and glutamate receptors are susceptible to volatile anaesthetics of clinical concentrations [6–8]. Among the nAChR subunits, the pentamer composed of homogenous α7 subunits is the most prevalent subunit in the mammalian brain [9]. Furthermore, α7nAChR can interact with NMDAR via a direct protein–protein interaction, and form a protein complex with NMDAR [10]. The α7nAchR-NMDAR coupling modulates and enhances NMDA receptors mediated currents in hippocampus [11]. Also, previous study reported that the desensitization of α7nAChR modulates cellular excitability and synaptic activity depends mostly upon the negative modulation of the GABAA receptor, as well as the positive modulation of the NMDA receptor [12]. N-methyl-D-aspartate (NMDA) receptors which are L-glutamate-gated ion channel receptors, play a critical role in mediating of synaptic signal transmission and plasticity in the central nervous system [13–14]. Furthermore, NMDAR activity is necessary for the consolidation of cognitive function [15–18]. The activation of α7nAChR not only enhances NMDAR modulated whole-cell currents, but also facilitates NMDAR-dependent LTP in the hippocampal neurons of rats [19]. However, whether α7nAChR and NMDAR are involved in sevoflurane impairment is unkown.

In our present study, we have evaluated the long-term effects of sevoflurane, exposure to neonatal rats, by evaluating the expression of α7nAChR and NMDAR, dendritic spine morphology of hippocampal neurons and the spacial working memory. Therefore, our aim is to further confirm whether the α7nAChR regulation of NMDAR is involved in the mechanism of sevoflurane’s effect on the cognitive function, and how α7nAChR regulates NMDAR.

## Materials and methods

### 1. Animals

Seven-day-old male Sprague-Dawley (SD) rats, weighing 10 ~ 15 g were provided by the Laboratory Animal Center of the Military Medical Science, Academy of the Chinese People′s Liberation Army. Newborn and mother rats were housed under controlled conditions on 12-h light/dark cycles with free access to food and water at room temperature (24 ~ 26 °C). All experimental procedures were approved by the Scientific and Ethics Committee of Tianjin Medical University (Tianjin, China) and conformed to the National Institutes of Health guide for the care and use of Laboratory animals (NIH Publications No.8023, revised 1978). Sodium pentobarbital was used as anaesthesia for sacrificing rats, and maximum effort was made to minimize suffering and to reduce the number of animals necessary to obtain valid results.

### 2. Animal model preparation

Seven-day-old SD rats were put into a small homemade transparent plastic box (W 30 cm x D 25 cm x H 10 cm), with soda lime in the bottom. Two small holes were drilled through the box. One hole was tube-connected to the sevoflurane vaporizer(Drager, America) to input oxygen and sevoflurane and the other was fitted to the exhaust vent connected to an S/5 anaesthetic gas monitor (Ohmeda, Detax company, America) to monitor the concentrations of sevoflurane, oxygen and carbon dioxide. The rats in the sevoflurane group (group S) and α7nAChR agonist PNU-282987 (Abcam, Britain) + sevoflurane group (group PS) inhaled 3% sevoflurane and oxygen delivered at a rate of 3 L/min for 6 hours, and the control group (group C) and the α7nAChR antagonist MLA (Abcam, Britain) group (group M) inhaled oxygen at a rate of 3 L/min for 6 hours. Because the stage of 7-day-old rat is just equal to human of full-term, and MAC of sevoflurane in neonates is 3.3 +/- 0.2% and in infants 1–6 months of age is 3.2 +/- 0.1% [20]. So we choose 3% sevoflurane in our study. Finally, pure oxygen was administered for 30 minutes. During this period of anaesthesia, a heating blanket was padded under the box, and a rectal temperature probe was inserted to control the temperature at 37°C, while maintaining the spontaneous breathing of the rats.

### 3. Experiment protocol

#### 3.1 Experiment protocol 1

To explore the long-term effects of sevoflurane on the morphology of dendritic spines and spatial working memory as well as the role of α7nAChR and NMDAR on sevoflurane’s negative effects, 7-day-old rats were randomly divided into four groups (n = 32 per group): group C, group S, group PS, and group M, treated, respectively, with 30% oxygen for 6 hours, 3% sevoflurane + 30% oxygen for 6 hours, 3% sevoflurane + 30% oxygen for 6 hours +PNU-282987 and 30% oxygen for 6 hours+ MLA. PNU and MLA were administered intraperitoneally. After treatment, the rats in each group were raised to 2 months old, and then deeply anaesthetized with 1% sodium pentobarbital (35mg kg^-1^). They were then dissected, and the hippocampi were removed. Western blot (n = 8 per group) was used to determine the expression of α7nAChR as well as the membrane, intracellular and total expression levels of NMDAR subunits. After being raised to 2 months old, four groups received Y-maze test (n = 16 per group) to evaluate the spatial working memory. Golgi-cox staining (n = 8 per group) was used to measure the dendritic spine density and length.

#### 3.2 Experiment protocol 2

The following protocol was utilized to examine in vitro whether the activation or inhibition of α7nAChR could change the morphology of dendritic spines, whether α7nAChR could regulate the expression of Src-family tyrosine kinase (SFK) and NMDAR, brains of the 7-day-old rats were removed to prepare coronal slices of 180 μm thickness and were randomly divided into 5 groups (n = 16 per group): group C, group S, group PS, group P, and group M. They were then incubated with normal Artificial Cerebral Spinal Fluid (ACSF), sevoflurane (3%) + ACSF, sevoflurane (3%) + PNU (final concentration 500 nmol/L), PNU + ACSF (final concentration 500 nmol/L), and MLA + ACSF (final concentration 10 nmol/L), respectively. After 6 hours of incubation, western blot was used to determine the expression of SFK, as well as the surface, intracellular and total expression of NR1, NR2A and NR2B, while Golgi-cox staining was used to measure the dendritic spine density and length.

To investigate whether SFK is the medium between α7nAChR and NMDAR, and the relationship between these receptors and dendritic spine morphology, brain samples from the 7-day-old rats were used to prepare coronal slices of 180 μm thickness and were randomly divided into 3 groups (n = 8 per group): group C, group P, group PP. The slices were then incubated with normal Artificial Cerebral Spinal Fluid (ACSF), PNU + ACSF (final concentration 500 nmol/L), and PNU (final concentration 500 nmol/L) + SFK inhibitor PP2 (Selleck, China, final concentration 10^4^ nmol/L) + ACSF, respectively. After 6 hours of incubation, western blot was used to determine the expression of the surface, intracellular and total expression of NR2B, and Golgi-cox staining was performed to measure the dendritic spine density and length.

Preparation of the hippocampus slices was performed as follows. The 7-day-old rats were deeply anaesthetized with 1% sodium pentobarbital (35 mg kg^-1^) and sacrificed to remove the brains. The latters were then iced for 5 min in 0°C ACSF A solution, bubbled with 95% O_2_ + 5% CO_2_ gas. ACSF A solution contains 235 mM Glucose, 2.5 mM KCl, 4 mM MgCl_2_, 1 mM CaCl_2_, 1.25 mM NaH_2_PO_4_, 26 mM NaHCO_3_, and 10 mM D-glucose, pH 7.4, with HCl and NaOH. The brain was sliced into 180-μm-thick portions by Leica VT1000S vibratome (Shimadzu, Japan) and then transferred to ACSF B solution + PNU/MLA, bubbled with 95% O_2_+5% CO_2_ gas, or ACSF B solution+ sevoflurane with 95% O_2_ + 5% CO_2_ gas at room temperature (22°C~24°C), while maintaining the concentration of sevoflurane at 3%. ACSF B solution contained 126 mM NaCl, 3 mM KCl, 2 mM MgCl_2_, 2 mM CaCl_2_, 1.25 mM NaH_2_PO_4_, 26 mM NaHCO_3_, and 10 mM D-glucose, pH 7.4, with HCl and NaOH. After incubation for 6 h, the hippocampus tissue was collected for western blot and Golgi-cox staining.

### 4. Y maze

Referring to the same methodology as in the previous literature [21], Y-maze test was used to evaluate the spatial working memory in rats aged 2 months. The apparatus (XR-XY1032, China) of the Y-maze test was randomly set as three arms, named A, B and C, within which the rats moved around freely for 8 minutes. An arm entry was defined as the entry of four paws into one arm. The order and number of entries the rats entered into each arm were recorded. The total number of entrances of each arm determined the general locomotor activity. The number of spontaneous alternation (one actual alternation was defined as entries into all three arms consecutively, i.e., ABC, BAC, or CAB but not BAB helped evaluate the spatial memory capacity. Visual cues made of white paper cut in different geometric forms were placed on the floor and at the end of each arm. To evaluate the spatial working memory without place preference, the appropriate visual cues for each arm were established in such a way that the rats did not show signs of avoidance or preference. The percentage of spontaneous alternations was defined according to the following equation: % Alternation = [(Number of alternations) / (Total arm entries − 2)] × 100. Data were eliminated in cases with fewer than 10 total arm entries.

### 5. Golgi-Cox staining

We used the same methodology as the one employed in a previous study [22]. Golgi-cox staining solution was prepared, and before use, it was stored for 1 day in the dark. Rats were guillotined after being anaesthetized with sodium pentobarbital. The brain was suspended in 100 ml of Golgi-Cox staining solution, and preserved for 14 days in the dark at 37°C. Then the brain was transferred into 30% sucrose solution, steeping for 2~5 days to increase its toughness. Brain coronal sections were sliced at 180 μm thickness by a Leica VT1000S vibratome. All the slices were collected, developed and fixed by the free-floating method. After being rinsed with distilled water for 10 minutes, the slices were mounted, dried, dehydrated, made transparent, and sealed. The images were observed under the microscope and collected. Dendritic spines from the apical dendrites of 20 hippocampal neurons in the stratum radiatum of the CA1 area were collected for analysis in each rat. The NIH Image analysis program version 1.43 was used to analyse the dendritic spine density and spine length of each neuron. The dendritic spine density is the number of dendritic spines per 10 μm on the secondary dendrites branch, and the dendritic spine length is the vertical dimension from the tip head of the dendritic spines to the dendritic shaft. Ten neurons were analysed in each rat.

### 6. Western blot

The rats were anaesthetized with sodium pentobarbital, and the hippocampi were removed after all experimental treatments. After homogenization in RIPA solution buffer (Solarbio, China), the hippocampal tissues were centrifuged at 4°C at 15,000 g for 10 minutes, and the supernatant was extracted as the total protein. The membrane and cytoplasmic proteins were extracted using a membrane compartment protein extraction kit (BioVision, America). The protein content was determined by a BCA protein assay kit (Well-bio, China). Protein samples of the same amount were separated by the method of sodium dodecyl sulfate–polyacrylamide gel electrophoresis (SDS–PAGE) and transferred to polyvinylidene difluoride filter membranes. The membranes were blocked by 5% nonfat milk in TBST for 60 min and then incubated with primary antibodies (rabbit α7nAChR diluted 1:500, Abcam; rabbit anti-NMDA-R1 diluted 1:1000, Abcam; rabbit anti-NMDA-R2A diluted 1:1000, Abcam; and rabbit anti-NMDA-R2B diluted 1:1000, Abcam) overnight at 4°C. After 5 washes with TBST for 5 minutes, the membranes were incubated with goat anti-rabbit (1:5000; Invitrogen, America) secondary antibodies for 1 h at room temperature. The membranes were washed another 5 times with TBST and then treated with a chemiluminescence detection kit (Millipore, America). The intensity of the protein bands was quantified by the Quantity One analysis software (Bio-Rad, Hercules, Canada). The relative expression of the proteins was expressed as a ratio to the expression of β-actin (diluted 1:4000, Abcam).

### Statistical analysis

The percentage of spontaneous alternations was considered the primary outcome. A power analysis was implemented to calculate the sample size based on our previous results. The mean percentage of spontaneous alternations of the four treatment groups (Group C, Group S, Group PS, and Group M) were 68%, 45%, 65%, and 55%, respectively. We determined a difference of at least 30% (error standard deviation = 18.0) among the treatment groups. An a priori algorithm was used to estimate the required sample size for analysis of variance (ANOVA). A sample size of 16 rats per group was found to be sufficient to detect a significant difference (α = 5%) with a statistical power (β-value) of 0.9 in Y maze test. Since we also need 8 rats for Western blot, and 8 rats for Golgi-cox staining, we considered increasing the sample size to 32 rats in total for each group. Statistical analysis was performed using the SPSS 20.0 software. Data from all experiments were expressed as the mean ± SD. Within multiple comparisons, we used ANOVA with a Turkey correction and Student’s t test to compare two groups. Rats in each group were sampled randomly from different litters. Our main hypothesis is as follows: H_0_, The population means of the spontaneous alternations and the dendritic spine density and length are same among treatment groups and control group. H_1_, The population means of the spontaneous alternations and the dendritic spine density and length are not all the same among treatment groups and control group. A *P* value <0.05 was considered statistically significant.

## Results

### 1. Sevoflurane induces cognitive deficits and dendritic spine loss

**Fig 1:**
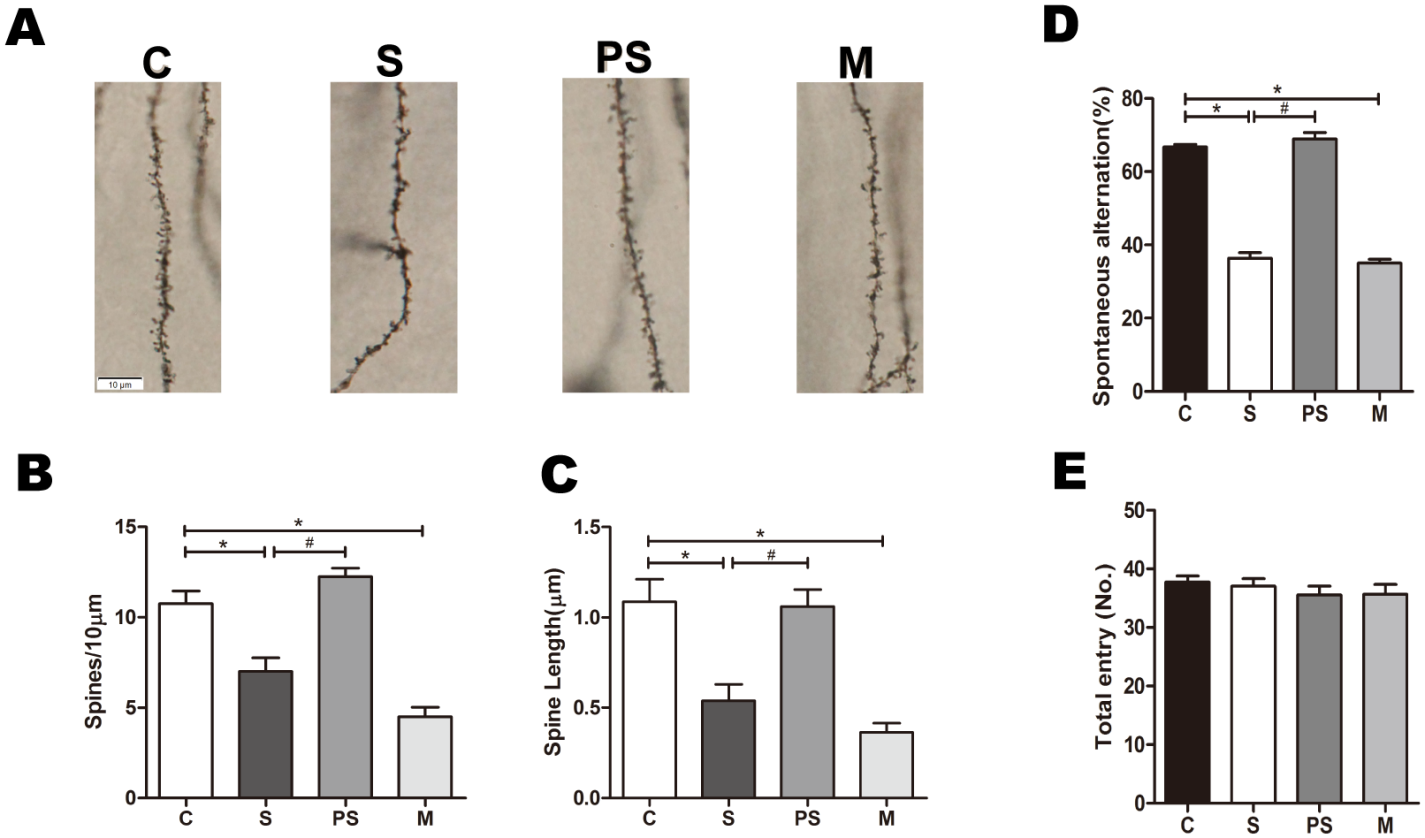
Sevoflurane impaired the dendritic spine morphology and memory function of 2-month-old rats. Golgi-Cox staining was performed for the dendritic spine density and length in stratum radiatum neurons of CA1 area in 2-month-old rats (A). Sevoflurane induced significant decreases in both dendritic spine density and length. n = 8 for each group; compared with group C, *P<0.01, ANOVA. α7nAChR agonist PNU-282987 prevented changes in dendritic spine density and length. n = 8 for each group; compared with group S, ^#^*P*<0.01, ANOVA. α7nAChR antagonist MLA induced significant decreases in dendritic spine density and length. n = 8 for each group; compared with group C, *P<0.01, ANOVA (B and C). In the Y-maze test (D), sevoflurane significantly decreased the percentage of spontaneous alternation in 2-month-old rats. n = 16 for each group; compared with group C, *P<0.01, ANOVA. α7nAChR agonist PNU-282987 prevented change in the percentage of spontaneous alternation. n = 16 for each group; compared with group S, ^#^*P*<0.01, ANOVA. α7nAChR antagonist MLA significantly decreased the percentage of spontaneous alternation. n = 16 for each group; compared with group C, *P<0.01, ANOVA. The total arm entries in Y-maze test were not different among the groups of 2-month-old rats (E).

To investigate whether exposing 7-day-old rats sevoflurane affects their cognitive function and dendritic spine morphology at 2 months of age, we used the method of spontaneous alternation in the Y-maze test to evaluate the spatial working memory and the Golgi-cox staining method to measure the dendritic spine density and length. In the Y-maze test, compared with group C, sevoflurane significantly decreased the percentage of spontaneous alternation in 2-month-old rats (Fig 1D, *P < 0*.*01*, n = 16 per group). The number of arm entries was not different among the groups (Fig 1E, *P > 0*.*05*, n = 16 per group), suggesting that although the ability of spatial memory was impaired, the capacity for action was not affected. In the Golgi-cox staining, we found that the dendritic spine density and length decreased in the population of rats that inhaled sevoflurane for 6 hours at 7 days of age, (Fig 1A, 1B and 1C, *P < 0*.*01*, n = 8 per group) compared with group C. These results indicate that sevoflurane alters the morphology of dendritic spines and thus lead to the possibilities of learning and memory deficits.

### 2. Sevoflurane decreases the expression level of α7nAChR and the membrane NMDAR subunits and inhibits the trafficking of NMDAR to the membrane

**Fig 2:**
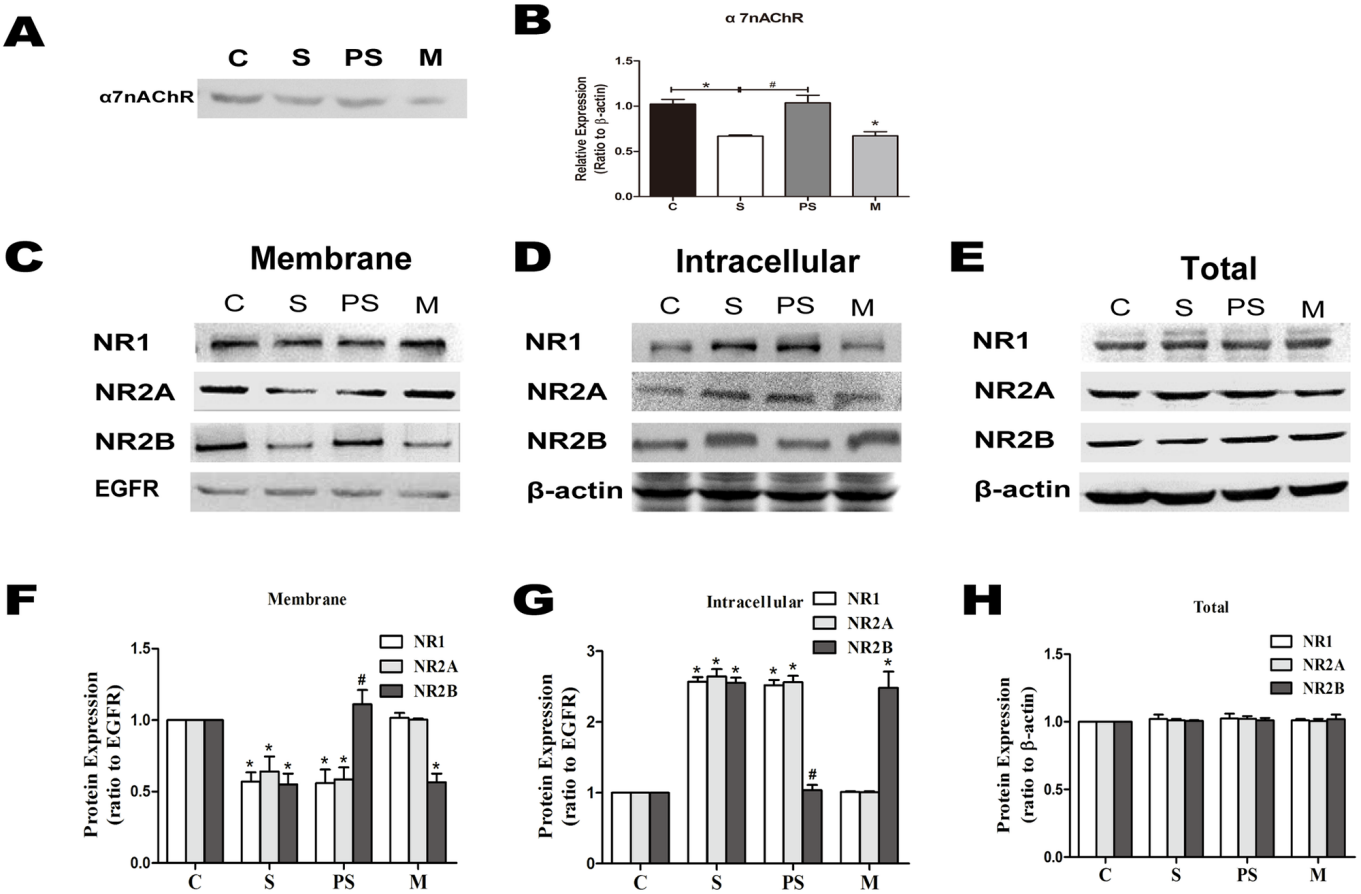
Sevoflurane decreased the expression levels of α7nAChR as well as the surface NMDAR containing NR1, NR2A and NR2B subunits in 2-month-old rat hippocampus. **PNU and MLA changed only the membrane expression of NMDAR-containing NR2B subunit**. Western blot for the expression of α7nAChR and the membrane, intracellular and total expression of NMDAR subunits was performed on 2-month-old rat hippocampus neurons (A, C, D and E). Epidermal growth factor receptor (EGFR) and β-actin were used as the loading controls. Pooled densitometric results for α7nAChR, NR1, NR2A and NR2B, with the band intensity of control group assigned the value of 1. Sevoflurane induced significant decreases of the expression level of α7nAChR. n = 8 for each group; compared with group C, *P<0.01, ANOVA. PNU-282987 prevented a change in the α7nAChR expression. n = 8 for each group; compared with group S, ^#^*P*<0.01, ANOVA. MLA significantly decreased the expression level of α7nAChR. n = 8 for each group; compared with group C, *P<0.01, ANOVA (B). Sevoflurane induced significant decreases in the membrane expression level of NMDAR subunits (NR1, NR2A and NR2B). n = 8 for each group; compared with group C, *P<0.01, ANOVA. PNU-282987 prevented a change in the membrane NR2B expression but not in that of NR1 or NR2A. n = 8 for each group; compared with group S, ^#^*P*<0.01, ANOVA. MLA significantly decreased the expression level of membrane NR2B but had no effect on the membrane protein levels of NR1 and NR2A. n = 8 for each group; compared with group C, *P<0.01, ANOVA (F). Sevoflurane induced significant increases in the intracellular expression level of NMDAR subunits (NR1, NR2A and NR2B). n = 8 for each group; compared with group C, *P<0.01, ANOVA. PNU-282987 prevented a change in the expression of intracellular NR2B, but not NR1 and NR2A. n = 8 for each group; compared with group S, ^#^*P*<0.01, ANOVA. MLA significantly increased the expression level of intracellular NR2B but had no effect on the intracellular protein levels of NR1 and NR2A. n = 8 for each group; compared with group C, *P<0.01, ANOVA (G). The total expression levels of NR1, NR2A and NR2B were not different among the groups (H).

Furthermore, we found that at 2 month old, the expression level of α7nAChR (Fig 2A and 2B, *P < 0*.*01*, n = 8 per group) and the membrane NMDAR subunits (NR1, NR2A and NR2B) were reduced by exposing 7-day-old rats to sevoflurane (Fig 2C and 2F, *P < 0*.*01*, n = 8 per group). However, the levels of intracellular NMDAR subunits (NR1, NR2A and NR2B) were increased by sevoflurane (Fig 2D and 2G, *P < 0*.*01*, n = 8 per group), and the total expression levels of NR1, NR2A and NR2B were not different among the groups (Fig 2E and 2H, *P > 0*.*05*, n = 8 per group). Together, these results show that sevoflurane induced the trafficking of NMDAR subunits (NR1, NR2A and NR2B) from the membrane to the intracellular space.

### 3. Activation of α7nAChR could reverse the effects of sevoflurane

To explore the role of α7nAChR in the effects of sevoflurane, we administered α7nAChR agonist before 7-day-old rats inhaled sevoflurane. α7nAChR agonist clearly reversed the decrease in α7nAChR induced by sevoflurane, and the α7nAChR antagonist decreased its expression (Fig 2A and 2B, *P < 0*.*01*, n = 8 per group). We found that the lowered spontaneous alternation induced by sevoflurane was reversed significantly by the α7nAChR agonist PNU (Fig 1D, *P <* 0.01, n = 16 per group) and that the dendritic spine density and length were increased by PNU compared to those of group S (Fig 1A, 1B and 1C, *P <* 0.01, n = 8 per group). However, PNU only increased the expression level of membrane NR2B-containing NMDAR (Fig 2C and 2F, *P <* 0.01, n = 8 per group) but decreased that of intracellular NR2B-containing NMDAR (Fig 2D and 2G, *P <* 0.01, n = 8 per group), that is, PNU enhanced its trafficking to the membrane but not that of NR1 or NR2A. Conversely, as shown in Figs 1 and 2, MLA significantly decreased the percentage of spontaneous alternation, the dendritic spine density and length, and the expression level of membrane NR2B-containing NMDAR. These results indicate that the activation of α7nAChR reversed sevoflurane induced cognitive deficits and dendritic spine loss and α7nAChR may be the molecular basis of sevoflurane neurotoxicity. Furthermore, we found that α7nAChR could not only regulate the expression of NR2B-containing NMDAR but that its activation could prevent sevoflurane-induced NR2B membrane trafficking in the hippocampus.

### 4. In vitro, sevoflurane decreases the dendritic spine density and length in hippocampus, and the activation of α7nAChR reverses this effect

**Fig 3:**
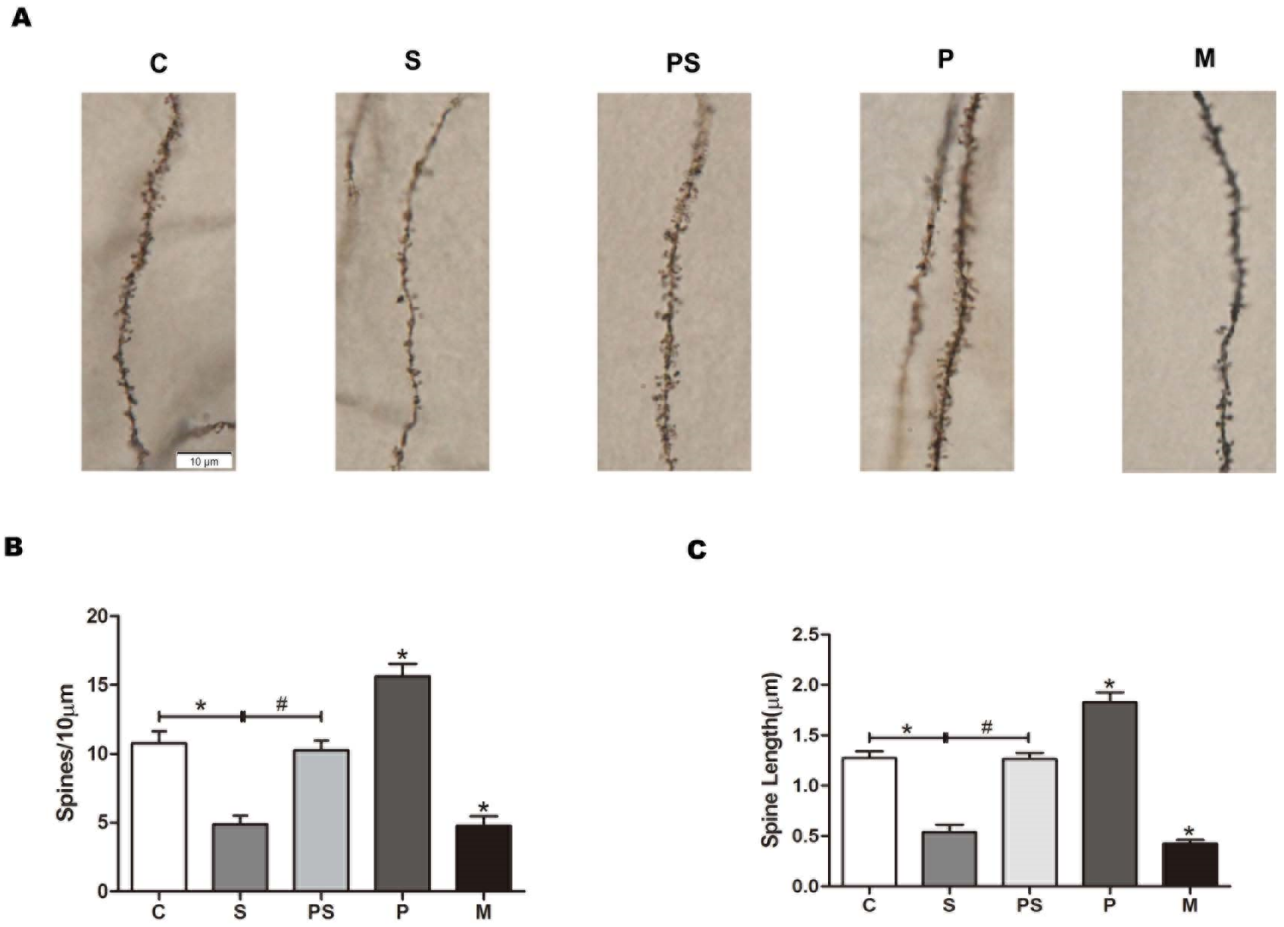
In vitro, sevoflurane and MLA clearly decreased the dendritic spine density and length, and PNU reversed this effect. Golgi-Cox staining was performed for the dendritic spine density and length in stratum radiaum neurons of CA1 area in 7-day-old rat brain slices (A). Sevoflurane induced significant decreases in dendritic spine density and length. n = 8 for each group; compared with group C, *P < 0.*01*, ANOVA. PNU-282987 prevented changes in dendritic spine density and length. n = 8 for each group; compared with group S, ^*#*^*P < 0*.*01*, ANOVA. PNU-282987 increased the dendritic spine density and length, but MLA induced significant decreases in dendritic spine density and length. n = 8 for each group; compared with group C, *P < 0.*01*, ANOVA (B and C).

To further verify the effect of sevoflurane on dendritic spine morphology and the role of α7nAChR therein, we incubated brain slices in ACSF. As shown in Fig 3A, 3B and 3C, the dendritic spine density and length were decreased after exposed to sevoflurane, but PNU reversed this effect (*P <* 0.01, n = 8 per group). In addition, compared with group C, PNU alone increased the dendritic spine density and length, whereas MLA had the opposite effect (*P <* 0.01, n = 8 per group). The data showed that in vitro, sevoflurane also induced dendritic spine loss, and the activation of α7nAChR reversed this effect, which is consistent with the in vivo results. Furthermore, the activation or inhibition of α7nAChR triggered changes in the dendritic spine density and length, which verified that α7nAChR could be a molecular basis of the dendritic spine changes.

### 5. In vitro, sevoflurane decreases the expression level of SFK and the membrane NMDAR. α7nAChR could regulate the expression levels of SFK and the membrane NR2B-containing NMDAR

**Fig 4:**
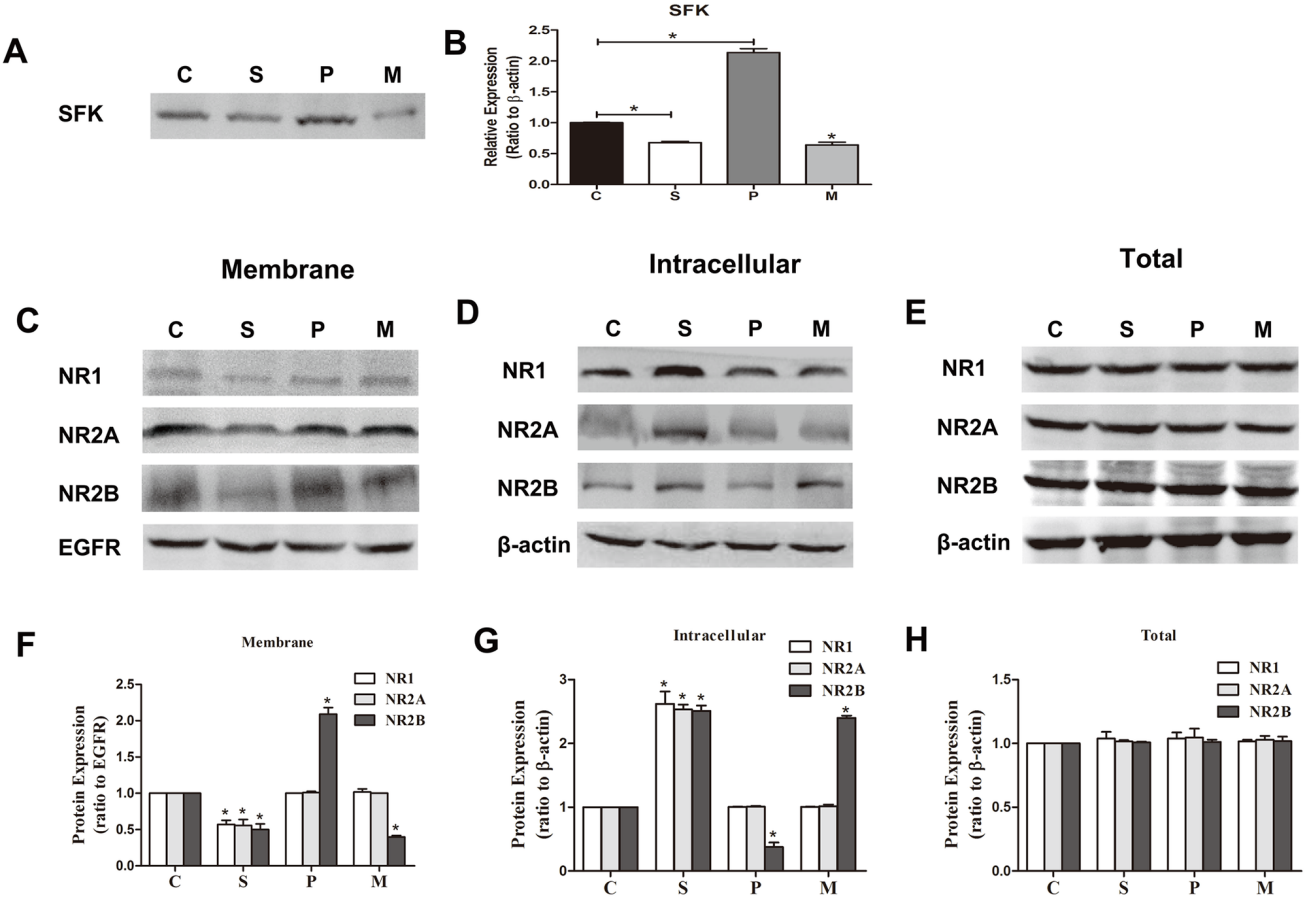
In vitro, sevoflurane decreased the expression of SFK and membrane NMDAR subunits. **PNU increased the expression level of SFK and the membrane NR2B subunit, and MLA had opposite effects**. Western blot for the expression of SFK as well as the membrane, intracellular and total expression of NMDAR subunits was performed on rat hippocampus neurons of 7-day-old rat brain slices (A, C, D and E). Epidermal growth factor receptor (EGFR) and β-actin were used as the loading controls. Pooled densitometric results for SFK, NR1, NR2A and NR2B, with the band intensity of control group assigned the value of 1. Sevoflurane induced significant decreases of the expression level of SFK. PNU-282987 increased SFK expression. MLA significantly decreased the expression level of SFK. n = 8 for each group; compared with group C, *P < 0.*01*, ANOVA (B). Sevoflurane induced significant decreases in the membrane expression level of NMDAR subunits (NR1, NR2A and NR2B). PNU-282987 increased the membrane NR2B expression but had no effect on the membrane protein levels of NR1 and NR2A. MLA significantly decreased the expression level of membrane NR2B but not of NR1 or NR2A. n = 8 for each group; compared with group C, *P < 0.*01*, ANOVA (F). Sevoflurane induced significant increases in the intracellular expression level of NMDAR subunits (NR1, NR2A and NR2B). PNU-282987 decreased the intracellular NR2B expression but had no effect on membrane protein level of NR1 and NR2A. MLA significantly increased the expression level of intracellular NR2B but not of NR1 or NR2A. n = 8 for each group; compared with group C, *P < 0.*01*, ANOVA (G). The total expression levels of NR1, NR2A and NR2B were not different among the groups (H).

As shown in Fig 4, sevoflurane decreased the expression levels of both SFK (Fig 4A and 4B, *P <* 0.01, n = 8 per group) and the membrane NMDAR subunits (Fig 4C and 4F, *P <* 0.01, n = 8 per group) and increased that of the intracellular NMDAR subunits (Fig 4D and 4G, *P <* 0.01, n = 8 per group). Meanwhile, SFK was increased by PNU but suppressed by MLA in the hippocampus (Fig 4A and 4B, *P <* 0.01, n = 8 per group). However, only the membrane NR2B-containing NMDAR was increased by PNU, compared to group C (Fig 4C and 4F, *P <* 0.01, n = 8 per group), and it was reduced by MLA (*P <* 0.01). The intracellular NR2B-containing NMDAR changed inversely (Fig 4D and 4G, *P <* 0.01), and the total expression of NMDAR subunits was not different among the groups (Fig 4E and 4H, *P >* 0.05). These results show that in vitro, sevoflurane induced NMDAR membrane trafficking in hippocampus, which is consistent with the in vivo results. In addition, we have shown in vivo that the activation of α7nAChR could prevent sevoflurane-induced NR2B membrane trafficking in the hippocampus. Here, in vitro, we found that the activation of α7nAChR increased NR2B trafficking from intracellular to membrane and that inhibiting α7nAChR decreased the membrane NR2B. That is, sevoflurane-induced NR2B membrane trafficking may act via α7nAChR regulation. Moreover, activation of α7nAChR increased the expression of SFK, and inhibition of α7nAChR had the opposite effect. These results suggest that α7nAChR, SFK and NR2B may have a relationship in this process.

### 6. α7nAChR may regulate NR2B-containing NMDAR and the morphology of dendritic spines via SFK

**Fig 5:**
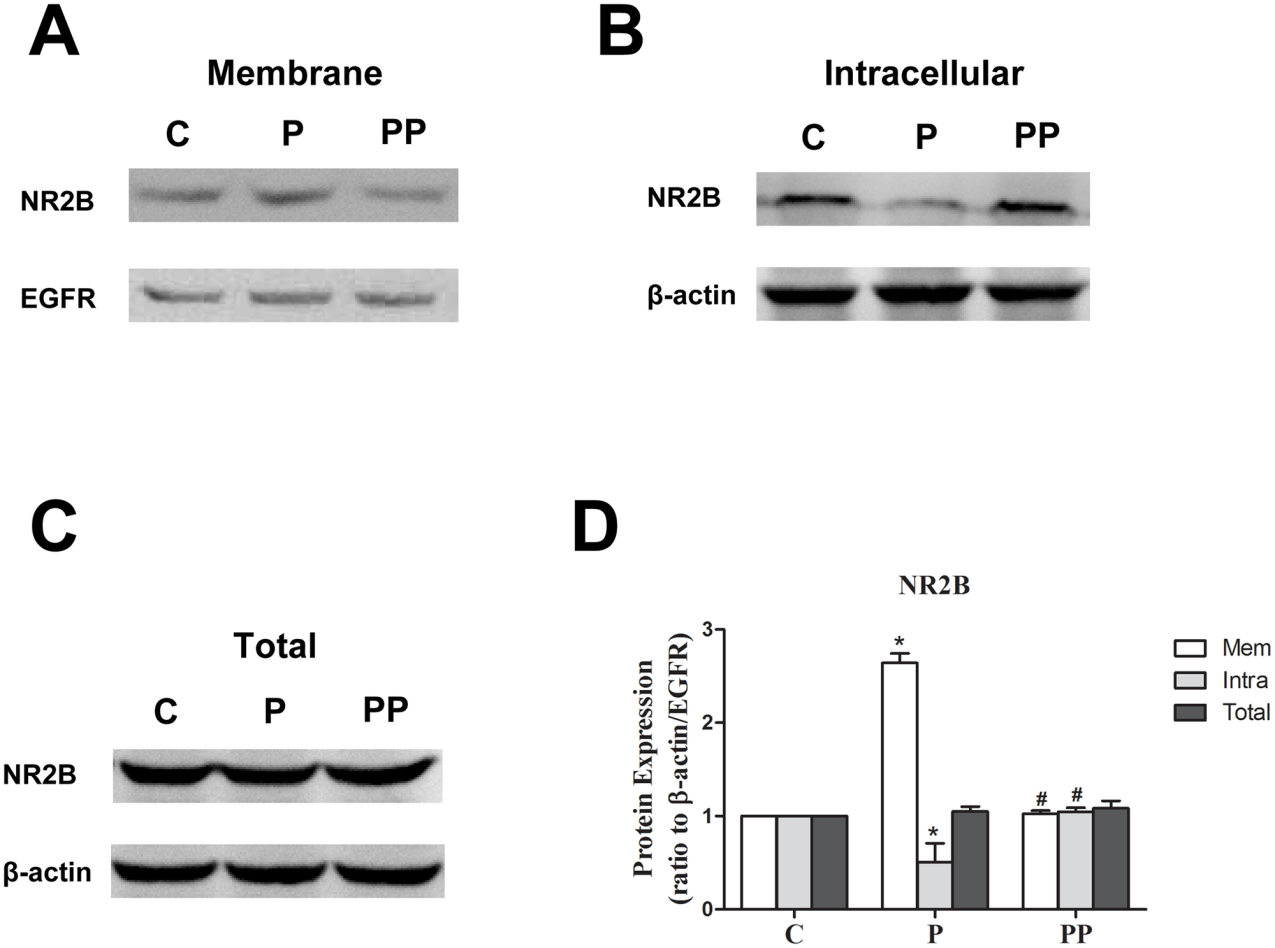
SFK inhibitor inhibited the upregulation of NMDAR-containing NR2B subunit induced by PNU. Western blot for the expression of the membrane, intracellular and total expression of NR2B was performed on rat hippocampus neurons from 7-day-old rat brain slices (A, B and C). Epidermal growth factor receptor (EGFR) and β-actin were used as the loading controls. Pooled densitometric results for NR2B, with the band intensity of control group assigned the value of 1. PNU-282987 significantly increased the membrane NR2B expression. n = 8 for each group; compared with group C, *P < 0.*01*, ANOVA. SFK inhibitor PP2 prevented changes in membrane NR2B expression. n = 8 for each group; compared with group P, ^*#*^*P < 0*.*01*, ANOVA (D). PNU-282987 significantly decreased the intracellular NR2B expression. n = 8 for each group; compared with group C, *P < 0.*01*, ANOVA. SFK inhibitor PP2 prevented changes in intracellular NR2B expression. n = 8 for each group; compared with group P, ^*#*^*P < 0*.*01*, ANOVA (D). The total expression levels of NR2B were not different among the groups (D).

**Fig 6:**
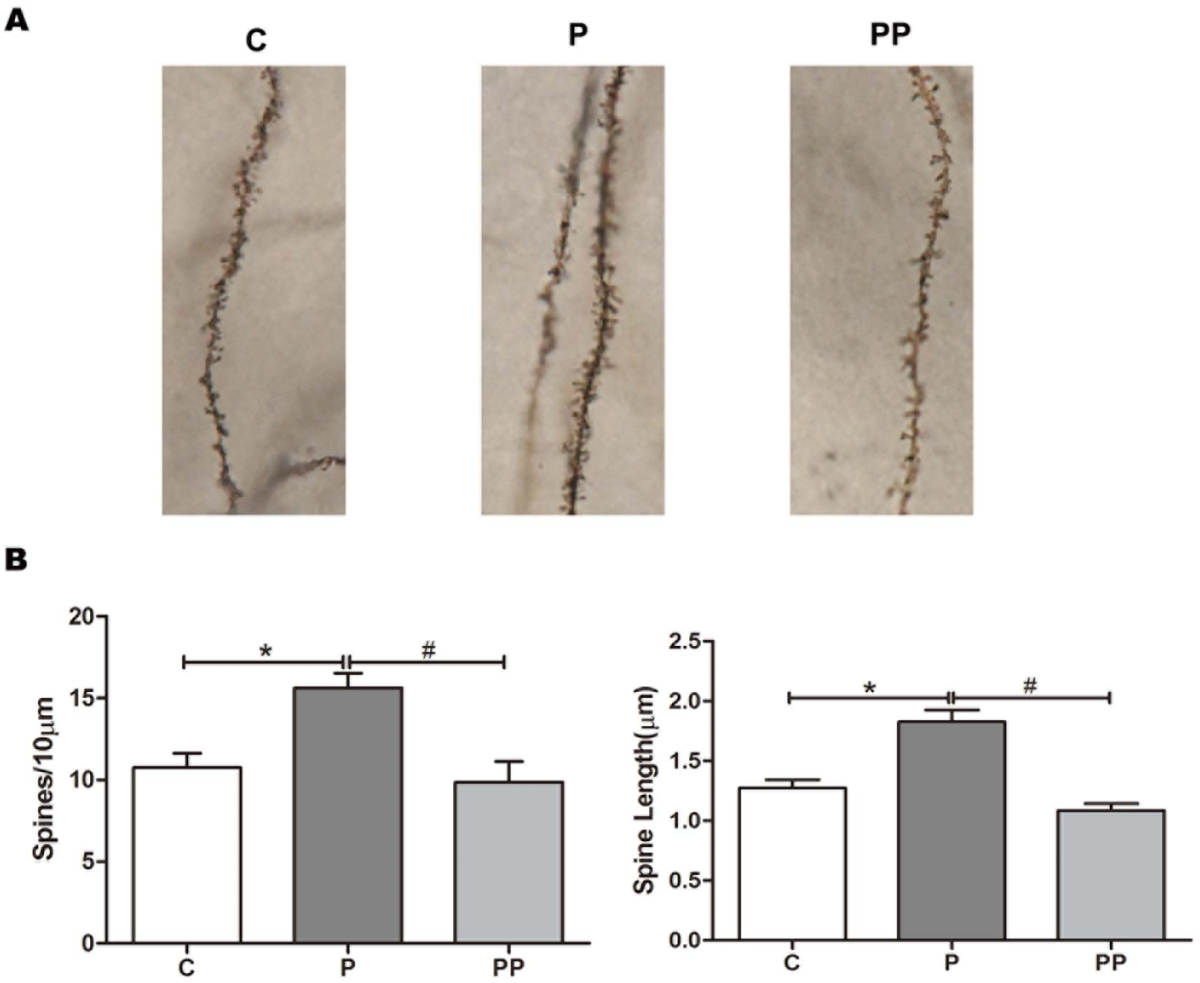
SFK inhibitor inhibited the increase in dendritic spine density and length induced by PNU. Golgi-Cox staining was performed for the dendritic spine density and length in stratum radiaum neurons of CA1 area on 7-day-old rat brain slices (A). PNU-282987 induced significant increases in dendritic spine density and length. n = 8 for each group; compared with group C, *P < 0.01, ANOVA. SFK inhibitor PP2 prevented changes in dendritic spine density and length. n = 8 for each group; compared with group P, ^#^*P* < 0.01, ANOVA (B).

To explore how α7nAChR may regulate NR2B-containing NMDAR, we applied the α7nAChR agonist PNU and SFK inhibitor PP2 in vitro. We found that SFK inhibitor inhibited the upregulation of the membrane NR2B-containing NMDAR induced by PNU (Fig 5A, *P <* 0.01, n = 8 per group), and increased the intracellular NR2B-containing NMDAR at the same time (Fig 5B, *P < 0*.*01*, n = 8 per group). The increase in dendritic spine density and length by PNU was also inhibited by PP2 (Fig 6A and 6B, *P <* 0.01, n = 8 per group). These results suggest that α7nAChR can regulate NR2B-containing NMDAR via SFK and that this molecular pathway may be involved in the changes in dendritic spine density and length.

## Discussion

In this study, we found that exposing neonatal rats to sevoflurane could weaken the ability of spatial memory at the age of 2 months and decrease the dendritic spine density and length of the neurons in the hippocampus. Furthermore, we showed that sevoflurane reduced the expression levels of α7nAChR and induced a significant increase in NMDA receptor subunits trafficking from the intracellular pool to the cell surface pool. More importantly, pretreatment with PNU, an α7nAChR agonist, could attenuate the development of sevoflurane-induced memory deficit and dendritic spine changes, which might occur by regulating NR2B-containing NMDAR trafficking from the intracellular pool to the cell surface pool in the hippocampus. Moreover, we demonstrated that α7nAChR may regulate NR2B-containing NMDAR via SFK and that the role of α7nAChR in the forming of dendritic spines has something to do with SFK signalling.

Recently, studies showed that sevoflurane, when given to 7-day-old rats, impaired early long-term memory and spatial memory in adulthood [23–24], which is consistent with our study. Prenatal morphine exposure exerted long-term decreased modulation on the expression of three NMDAR subunits (NR1, NR2A, and NR2B) within the hippocampal CA1 subregion, resulting in rat offspring exhibiting impaired performances in the spatial working and cued reference memory [25]. Here, in our study, neonatal sevoflurane exposure also had decreased the expression of three NMDAR subunits (NR1, NR2A, and NR2B) in 2 month old rat hippocampus. In additon, our study indicated that sevoflurane induced changes in dendritic spine morphology. As is known, dendritic spines receive the synaptic signalling of most excitatory synapses in the CNS, where they play an important role in the transmission and integration of messages among synapses [26]. However, exposing to postnatal day 5 ~ 7 mice to isoflurane for 2 hours reduced the number of dendritic filopodial spines and synapses of hippocampal neurons in mice [27]. This could lead to subsequent cognitive disorders in adulthood. α7nAChRs, which are targeted by volatile anaesthetics, are located on almost all synapses and bodies or processes of hippocampal neurons at CA1 stratum radiatum [28]. Lowered glutamatergic synapses and synaptic deficits are present in mice that lack the α7nAChR gene, which suggests that α7nAChR is essential to retain the normal number and function of synapses [29]. In addition, the number of immature dendritic spines in the CA1 area of hippocampus increases by 64% in α7nAChR knockout mice, with a significant decline in branching. This finding indicates that α7nAChR could promote the dendritic spines to a mature state, which is closely related to normal learning and memory abilities [30]. NMDAR subunits (NR2A and NR2B) cluster on the dendritic spines, and their expression level is parallel to the formation of dendritic spines and synapses [31]. However, during the 2 weeks following birth in the rat hippocampus, there is a developmental switch of NMDARs from containing the NR2B subunit to containing the NR2A subunit [32]. More importantly, the interaction of NR2B with CaMKII may lead to the formation of new spines and synaptic contacts [33]. The activation of integrins could affect the morphology of dendritic spines, such as by prolonging the existing dendritic spines and forming new filopodia through activation of NMDAR [34], indicating that NMDAR activation may facilitate the growth of dendritic spines. Moreover, an approximately 50% blockade of the NMDARs has shown to cause a decline in spine density [35]. This may be due to the inhibition of NMDARs which reduces Ca^2+^ influx via NMDAR and then weakens the NMDAR-induced long-term potentiation (LTP). Since NMDAR-dependent LTP could enhance the formation of new spines [36], the dendritic spines will decline when NMDARs are blocked. The dendritic spine density was decreased in CA1 pyramidal neurons in mice lacking the NR2B gene, but the spine length was not different from that of the wild type [37]. This result demonstrates that the inhibition of all three NMDAR subunits in our experiment probably led to an increasing induction of LTD and then gave rise to the shrinkage of dendritic spines [38]. Overall, the morphological alteration of dendritic spines induced by sevoflurane may be associated with the function of α7nAChR and NMDAR, and among the NMDAR subunits, NR2B plays a more important role.

Sevoflurane reduces the activation of nAChR at anaesthetic concentrations [39]. Moreover, sevoflurane could give rise to caspase activation with an increased accumulation of β-amyloid protein in neonatal mice [40]. Then the accumulation of Aβ causes NMDAR internalization [41], which inhibits NMDAR trafficking to the membrane. Therefore, sevoflurane may inhibit NMDAR indirectly. In addition, other mechanisms of sevoflurane regulating NMDAR have been reported. Sevoflurane-induced neuronal apoptosis was attenuated by synaptic NMDA receptors activation, which restored the sevoflurane-induced phospho-ERK1/2 inhibition. The neuroprotective role of synaptic NMDA activity could be reversed by the MEK1/2 inhibitor U0126 in vitro. Therefore, ERK1/2 MAPK signalling may be involved in the relief of sevoflurane neurotoxicity by NMDA receptors [42]. Besides, sevoflurane exposure increases MeCP2 (methyl-CpG island binding protein 2) phosphorylation in the hippocampus of 7-day-old mice [43]. Chromatin immunoprecipitation (ChIP) assays demonstrated that two of the five NR2B promoter area sites bind MeCP2. The activity-dependent expression of NMDA subunit (NR2B) is mediated by MeCP2-dependent epigenetic regulation [44]. Therefore, sevoflurane may also regulate NMDAR via MeCP2.

Both α7nAChR and NMDAR are ligand-gated ion channel receptors with high Ca^2+^ permeability. Co-immunoprecipitation experiments confirmed that in the hippocampus, α7nAChR and NMADR formed protein complex by direct interaction [10]. In the dorsolateral prefrontal cortex, the activation of α7nAChR can enhance the opening of the ion channels on NR2B-containing NMDARs, which plays an important role in the production of working memory mediated by α7nAChR [45]. Studies have shown that the activation of nAChR by nicotine in rat hippocampal CA1 area could increase the level of tyrosine phosphorylation of NR2B, but not NR1 or NR2A subunit of NMDAR [46]. In addition, NMDAR tyrosine phosphorylation can increase NMDAR membrane expression [47], and an increase in surface NMDAR can strengthen the function of NMDAR, consequently increasing the number of dendritic spines and enhancing the capacity of spatial working memory. Given these reasons, we speculate that the positive effect of α7nAChR activation on the dendritic spine loss and spatial working memory deficits caused by sevoflurane may be achieved by increasing NMDAR tyrosine phosphorylation, especially that of the NR2B subunit.

Src-family tyrosine kinase (SFK) inhibitor PP2 could suppress the trafficking of NR2B-containing NMDAR to the membrane [47]. Therefore, the upregulation of NMDAR induced by the activation of α7nAChR may be associated with SFK. The activation of SFK could enhance NMDAR mediated LTP in CA1 area of hippocampus, leading to a reduction of the intracellular NMDAR expression levels [48]. That is, the NMDAR expression level on the membrane is increased. Therefore, the tyrosine phosphorylation of NMDAR subunits regulated by SFK could play a role in stabilizing NMDAR on the membrane and increasing the function of NMDAR. In our study, when the inhibitor of SFK was administered, the upregulation of NR2B-containing NMDAR via the activation of α7nAChR was blocked. Therefore, we considered that α7nAChR may meditate NMDAR via the SFK pathway.

In conclusion, the present study suggests that the inhibition of α7nAChR contributes to sevoflurane-induced cognitive deficits and dendritic spine loss by regulating NR2B-containing NMDAR trafficking in the developing rat hippocampus and that α7nAChR regulation of the trafficking of NR2B-containing NMDAR via SFK pathway may underlie the mechanism of sevoflurane effects on the brain neurons. The present work may shed new light on inhalational anaesthetic neurotoxicity and, more practically, on developing new drugs to target the clinical prevention and treatment of negative effects of sevoflurane on the developing brain.
